# Effects of Grain Shape Genes Editing on Appearance Quality of Erect-Panicle *Geng/Japonica* Rice

**DOI:** 10.1186/s12284-021-00517-5

**Published:** 2021-08-10

**Authors:** Ting Mao, Mingdong Zhu, Zhonghua Sheng, Gaoneng Shao, Guiai Jiao, Amos Musyoki Mawia, Shakeel Ahmad, Lihong Xie, Shaoqing Tang, Xiangjin Wei, Shikai Hu, Peisong Hu

**Affiliations:** 1 State Key Laboratory of Rice Biology, China National Center for Rice Improvement, China National Rice Research Institute, Hangzhou, 310006 China; 2 Liaoning Institute of Saline-Alkali and Utilization, Panjin, 124010 China; 3 Hunan Rice Research Institute, Changsha, 410125 China

**Keywords:** Rice, Grain shape gene, Appearance quality, Gene editing, Molecular design breeding

## Abstract

Long grain *geng/japonica* rice has a higher market preference due its excellent appearance quality. The *dense and erect-panicle 1* (*dep1*) gene has been widely used in the breeding of high-yielding *geng/japonica* rice cultivars in China. However, this gene causes short and round grain shape thus making it less attractive in global rice markets. Therefore, breeding of high-yielding long-grain *geng/japonica* rice cultivars by incorporating *dep1* with major-effect grain shape gene is of high priority in rice industry. Up to now, multiple grain shape genes’ effect mechanism has been clearly elaborated, however, under the background of erect-panicle *geng/japonica* rice, the effect of major grain shape genes on the appearance quality need to be further clarified, as detailed reports are limited. Utilizing CRISPR/Cas9 technology, a series of near-isogenic lines (NILs) (YF47^*dep1*^-*gw8*, YF47^*dep1*^-*gs3*, YF47^*dep1*^-*gl7*, YF47^*dep1*^-*qgl3* and YF47^*dep1*^-*tgw6*) in Yanfeng 47(YF47^*dep1*^) background were created. Grain appearance and yield components analysis showed that: (1) All NILs’ grain length to width ratio was significantly increased compared to that of YF47^*dep1*^, excepted YF47^*dep1*^-*gs3*, (2) The chalkiness degree was significantly reduced in all of the NILs, (3) In all of the NILs, YF47^*dep1*^-*gw8* grains exhibited the greatest length to width ratio and the lowest chalkiness degree, (4) The composition of glume cells and filling characteristics of the endosperm were two key factors contributing grain shape and grain chalk variations, respectively, and **v)** Owning to a substantial increase in the thousand grain weight, the yields of YF47^*dep1*^-*gs3* and YF47^*dep1*^-*tgw6* were significantly higher than that of YF47^*dep1*^, whereas YF47^*dep1*^-*qgl3* exhibited the lowest yield because of a dramatic decrease in the effective panicle number and thousand grain weight. All the results revealed that pyramiding *dep1* with major-effect grain shape alleles was an effective approach to improving the appearance quality of erect-panicle *geng/japonica* rice, owning to both of the appearance quality and yield improvement, *GS3* and *TGW6* alleles can be applied directly for breeding long-grain shape *geng/japonica* rice, and editing *GW8* resulted in excellent appearance quality but low yield, therefore, this gene would be difficult to use directly but can be considered as the core germplasm resource.

**Supplementary Information:**

The online version contains supplementary material available at 10.1186/s12284-021-00517-5.

## Findings

With the structural adjustment and upgrading of the rice industry, the market demand for superior quality *geng/japonica* rice is increasing every year (Chen et al. 2018). The grain shape of traditional *geng/japonica* rice in China is mostly short and round, but recently long grain shape has become preferred by the market because of its excellent appearance quality (Xu and Chen 2016; Huang and Qian 2017). Currently, the *dep1* gene is widely used in the breeding of *geng/japonica* rice in China due to high-yielding population characteristics, however, the gene also brings in characteristics of short grains and low thousand grain weight (Liu et al. 2018). Therefore, breeding of high-yielding long-grain *geng/japonica* rice cultivars by incorporating *dep1* with major-effect grain shape genes is of high priority in rice industry, as the theoretical basis and required germplasm reports are limited (Chen et al. 2012; Xu and Chen 2016).

Multiple genes for grain shape have been cloned, some of which have strong effects and wide applications, including the major-effect genes controlling grain length, *GS3*, *qGL3* and *GL7*/*GW7*, the thousand grain weight gene *TGW6*, plant shape gene *DEP1* (Fan et al. 2006; Zhang et al. 2012; Ishimaru et al. 2013; Wang et al. 2015a, b) and major-effect genes that control grain width, *GW2*, *GW5*, *GS5* and *GW8* (Song et al. 2007; Wan et al. 2008; Li et al. 2011; Wang et al. 2012). Multiple studies have shown that the grain shape genes directly determine the shape of rice grains by regulating the development of glume cells (Fan and Li 2019), and also indirectly affect grain chalk by determining endosperm development after fertilization (Wang et al. 2008; Li et al. 2014). In addition, a series of endogenous hormones and starch synthesis-related enzymes are also involved in the dynamic development of the glumes and endosperm. For example, brassinosteroids (BR) and indole acetic acid (IAA) are reportedly related to the development of glume cells (Li et al. 2018), whereas the enzyme activities of adenosine diphosphate glucose (ADPG), granule‐bound starch synthase (GBSS), soluble starch synthase (SSS) and starch branching enzyme (SBE) on endosperm development is significant (Dong et al. 2008).

In the present study, utilizing CRISPR/Cas9 technology, we created a series of NILs (YF47^*dep1*^-*gw8*, YF47^*dep1*^-*gs3*, YF47^*dep1*^-*gl7*, YF47^*dep1*^-*qgl3* and YF47^*dep1*^-*tgw6*) in YF47^*dep1*^ background. Further, the comparison of grain appearance and yield were contracted, and the related impact factors were also deeply clarified. The objectives of this study included: (1) Clarifying the effects of *GW8*, *GS3*, *GL7*, *qGL3*, and *TGW6* genes (partial major-effect grain shape genes) on grain appearance and yield of erect panicle rice, (2) creating a series of long-grain erect-panicle *geng/japonica* rice gremplasm, and evaluating its breeding application value in aspect of appearance quality and yield, and (3) providing the theoretical basis for breeding long-grain shape *geng/japonica* rice.

**Fig. 1:**
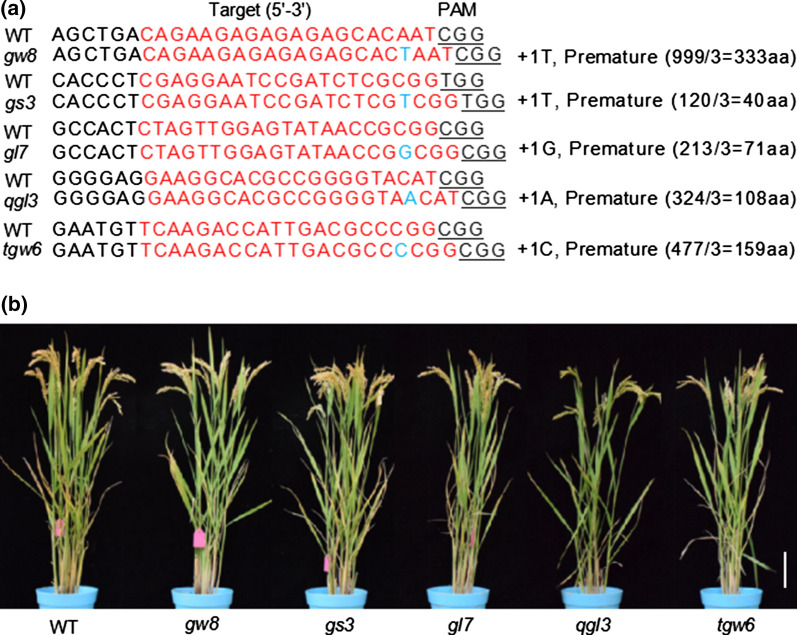
The target sequence, resulting amino acid changes and plant architecture of the constructed NILs. **a** The mutation in the target DNA and amino acid sequences. The red and blue nucleotides represent target sequences and inserted mutation, respectively, and the protospacer adjacent motif (PAM) sequences are underlined. **b** Representative plant architecture of the NILs. Scale bar = 10 cm

We analyzed YF47^*dep1*^ and 96 *geng/japonica* rice varieties’ genotype distribution at the *GW8*, *GS3*, *GL7*, *qGL3* and *TGW6* loci, which were widely used in *xian/indica* rice breeding programs. As shown in Additional file 3: Fig. S1 and Additional file 2: Table S1, YF47^*dep1*^ was classified as short and round grain type (“Nipponbare” type) on both of the above-mentioned locus, and also, very few long grain type alleles have been used in *geng/japonica* rice breeding in northern China. Therefore, by utilizing CRISPR/Cas9 technology, we created a series of NILs as our research materials, which carried the mutant alleles at the above loci under the background of *geng/japonica* rice cultivar, YF47^*dep1*^, having an erect panicle architecture. The NILs comprised of YF47^*dep1*^-*gw8*, YF47^*dep1*^-*gs3*, YF47^*dep1*^-*gl7*, YF47^*dep1*^-*qgl3* and YF47^*dep1*^-*tgw6*, which present single base insertion in target site and led to truncated proteins of various sizes compared with YF47^*dep1*^ (wild type, WT) (Fig. 1a). Besides the number of tillers, the plant architecture of NILs had insignificant differences compared with YF47^*dep1*^ (Fig. 1b).

**Fig. 2:**
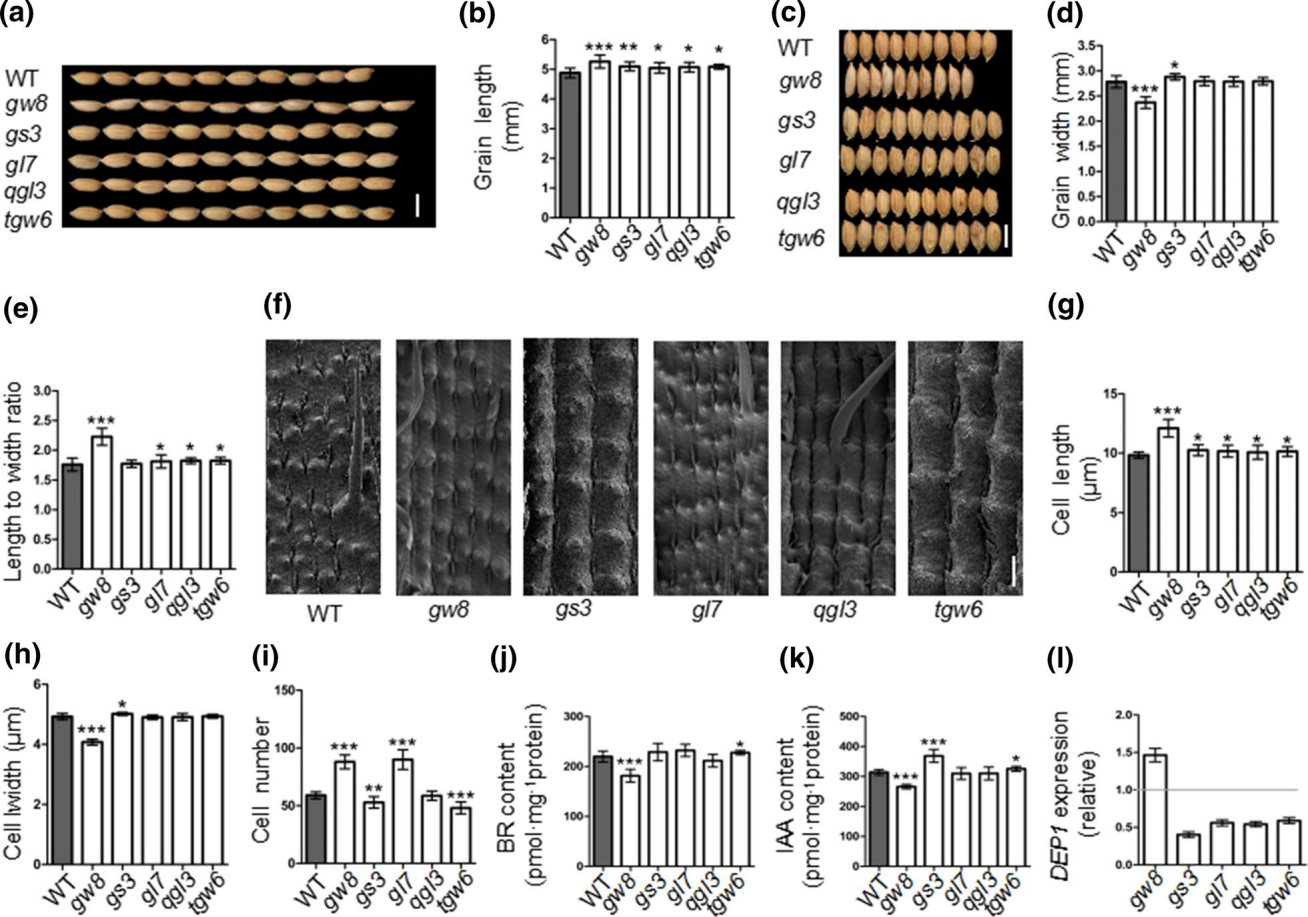
The appearance and determination of grain shape and related impact factors analysis in YF47^*dep1*^ (WT) and the NILs. **a**, **b** The appearance (**a**) and comparison (**b**) of grain length in WT and NILs. Scale bar = 5 mm. The data represent the mean ± sd, ***P < 0.001, **P < 0.01, *P < 0.05, the same as below. **c**, **d** The appearance (**c**) and comparison (**d**) of grain width in WT and NILs. Scale bar = 5 mm. **e** The comparison of length to width ratio in WT and NILs. **f–i** The appearance (**f**) and comparison of cell length (**j**), cell width (**h**) and number of cells (**i**) on the outer surface of the glumes in WT and the NILs. Scale bar = 10 μm. **j**, **k** The comparison of IAA (**j**) and BR (**k**) levels in YF47^*dep1*^ (WT) and the NILs. **l** The relative expression of *DEP1* in the NILs

The grain lengths and widths were determined at maturity stage (Fig. 2a–d), the grain length of YF47^*dep1*^-*gw8* was 5.29 mm and around 5.1 mm for the other NILs, which showed significant increase compared to that of YF47^*dep1*^(4.9 ± 0.08 mm). The grain width was significantly lower in YF47^*dep1*^-*gw8* (2.39 ± 0.09 mm) and significantly higher in YF47^*dep1*^-*gs3* (2.90 ± 0.06 mm), compared to YF47^*dep1*^ (2.80 ± 0.09 mm). No significant differences in width were observed in the other lines. The average length to width ratio of YF47^*dep1*^ was 1.74 (Fig. 2e), but significantly higher in YF47^*dep1*^-*gw8* (2.22 ± 0.11), YF47^*dep1*^-*gl7* (1.81 ± 0.11), YF47^*dep1*^-*qgl3* (1.84 ± 0.04), and YF47^*dep1*^-*tgw6* (1.82 ± 0.05). The length to width ratio of the YF47^*dep1*^-*gs3* grain was statistically the same as that of YF47^*dep1*^ due to the increase in its width. Scanning electron microscope analysis showed that the mean length of the glume cells in all of the NILs was significantly greater than that of YF47^*dep1*^, with those of YF47^*dep1*^-*gw8* being the longest (Fig. 2f–i). The cell width in YF47^*dep1*^-*gw8* was significantly reduced but substantially increased in YF47^*dep1*^-*gs3*, and no significant difference was observed in the other NILs. The cell number in YF47^*dep1*^-*gw8* and YF47^*dep1*^-*gl7* was significantly increased, whereas it decreased dramatically in YF47^*dep1*^-*gs3* and YF47^*dep1*^-*tgw6*.

It has previously been reported that increased levels of IAA and BR might increase fruit size (Li et al. 2018). Therefore, we analyzed the levels of the endogenous hormones IAA and BR during the most vigorous period of glume development (Fig. 2j–k). Of all the NILs, no significant change was seen in YF47^*dep1*^-*gl7* and YF47^*dep1*^-*qgl3*. However, the levels of IAA and BR were substantially reduced and elevated in YF47^*dep1*^-*gw8* and YF47^*dep1*^-*tgw6*, respectively. The IAA level was significantly elevated in YF47^*dep1*^-*gs3*, whereas its level of BR remained statistically the same although slightly elevated. Along with the thousand grain weight (TGW) performance in Table 1, we speculated that IAA and BR might promote the TGW by regulating glume development. In erect-panicle varieties, short and round grains with low TGW are prone to form because of the up-regulated expression of *DEP1* (Sun et al. 2018). In our study, apart from YF47^*dep1*^-*gw8*, the level of *dep1* expression was down-regulated in all of the transgenic plants (Fig. 2l), which may serve as a contributing factor to their elongated grain lengths. On the contrary, the level of *dep1* was increased in YF47^*dep1*^-*gw8*, which may be related to its significantly decreased TGW (Table 1).

**Table 1:** The comparison of yield traits in WT and the NILs

Varieties	NEP		NFGP		TGW (g)		Yield (kg ha^−1^)	
	2019	2020	2019	2020	2019	2020	2019	2020
YF47^*dep1*^ (WT)	18 ± 1.62	18 ± 1.15	132 ± 3.92	131 ± 2.12	25.9 ± 0.51	25.8 ± 0.65	10,132.84 ± 99.25	9860.48 ± 97.87
YF47^*dep1*^-*gw8*	19 ± 1.58	18 ± 0.87	128 ± 4.15**	125 ± 3.33**	23.2 ± 0.48**	23.1 ± 0.38**	9418.59 ± 112.14**	9168.47 ± 112.14**
YF47^*dep1*^-*gs3*	18 ± 1.34	18 ± 1.36	125 ± 2.98**	123 ± 3.15**	27.5 ± 0.39**	27.6 ± 0.29**	10,777.61 ± 86.51**	10,494.13 ± 115.21**
YF47^*dep1*^-*gl7*	19 ± 1.87	17 ± 0.97	127 ± 3.14**	129 ± 2.97*	24.4 ± 0.42**	24.3 ± 0.42**	9610.35 ± 79.24**	9287.97 ± 96.21**
YF47^*dep1*^-*qgl3*	16 ± 0.98**	15 ± 1.15**	131 ± 3.61	127 ± 3.45*	23.9 ± 0.48**	23.9 ± 0.61**	9085.29 ± 96.51**	8992.38 ± 97.89**
YF47^*dep1*^-*tgw6*	18 ± 1.12	19 ± 1.71	128 ± 3.28*	120 ± 4.12**	26.7 ± 0.54**	26.6 ± 0.42**	10,774.82 ± 115.41**	10,402.42 ± 93.21**

NEP, number of effective panicles; NFGP, number of filled grains per panicle; TGW, thousand grain weight; **P < 0.01, *P < 0.05

**Fig. 3:**
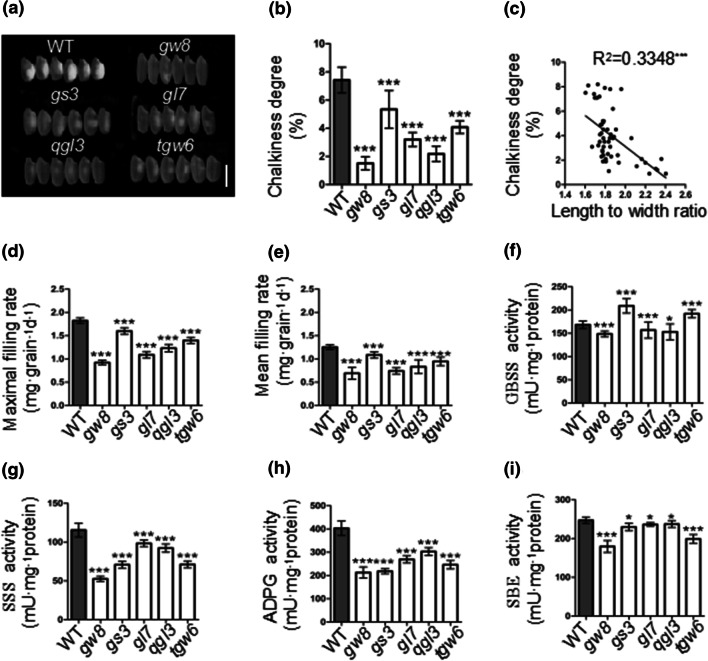
The appearance and determination of grain chalk and related impact factors analysis in YF47^*dep1*^ (WT) and the NILs. **a**, **b** The appearance (**a**) and comparison (**b**) of chalkiness degree in WT and the NILs. Scale bar = 5 mm. **c** The correlation analysis of the length to width ratio and chalkiness degree in YF47^*dep1*^ and the NILs. **d**, **e** The maximum (**d**) and average (**e**) grain filling rates in WT and the NILs. **f–i** The comparison of ADPG content (**f**), GBSS content (**g**), SSS content (**h**) and SBE content (**i**) in WT and the NILs during endosperm development

The grain chalky characteristics of the NILs were investigated after the grains were processed into polished rice (Fig. 3a, b). Chalkiness degree in all the NILs was significantly lower than that of YF47^*dep1*^, which demonstrated an average of 7.31%. The lowest level of chalkiness degree was observed in YF47^*dep1*^-*gw8* with a value of 1.51 ± 0.41%, followed by YF47^*dep1*^-*qgl3* (2.28 ± 0.57%) and YF47^*dep1*^-*gl7* (3.29 ± 0.51%). The chalkiness degree of YF47^*dep1*^-*gs3* and YF47^*dep1*^-*tgw6* was relatively high, measured as 5.37 ± 1.19% and 4.08 ± 0.40%, respectively. Also, the length to width ratio and chalkiness degree showed a significant negative correlation (Fig. 3c). This indicated that increasing the length to width ratio of grains and reducing chalkiness degree were harmonized in the erect-panicle background.

To further investigate the effect of endosperm development on grain chalkiness variations, we analyzed the grain filling rate by calculating the changes in endosperm weight during 0–35 days after flowering (Fig. 3d, e). The maximum and average grain filling rates for all the NILs were significantly reduced compared to that of YF47^*dep1*^. Of these, YF47^*dep1*^-*gw8* showed the lowest maximum and average grain filling rates, whereas the measurements for YF47^*dep1*^-*gs3* and YF47^*dep1*^-*tgw6* were relatively high. Wang et al., (2012) demonstrated that the activities of ADPG, GBSS, SSS and SBE are closely related to the filling rate of endosperm. Furthermore, we analyzed the activity changes in the starch biosynthesis-related enzymes in the NILs. Compared to YF47^*dep1*^, the activities of these enzymes were significantly reduced in all of the NILs (Fig. 3f–i), with the exception that the activity of GBSS in YF47^*dep1*^-*gs3* and YF47^*dep1*^-*tgw6* was substantially elevated. Of all the NILs, the activity levels of the four enzymes in YF47^*dep1*^-*gw8* were the lowest. These results indicated that the up-regulation of these four enzymes was helpful in increasing the filling rate, and the relatively high activity of GBSS in YF47^*dep1*^-*gs3* and YF47^*dep1*^-*tgw6* might be due to the high endosperm dry weight and high TGW.

The variation in yield traits of the NILs was compared in two consecutive growing seasons (Table 1). Generally, the yields of YF47^*dep1*^-*gs3* and YF47^*dep1*^-*tgw6* improved significantly compared to the control YF47^*dep1*^ due to the substantially increased TGW, whereas YF47^*dep1*^-*qgl3* exhibited the lowest yield due to the significantly reduced effective panicle number and TGW. However, the yields of YF47^*dep1*^-*gw8* and YF47^*dep1*^-*gl7* were relatively low owing to the dramatically decreased TGW and grain number per panicle.

While ensuring the high yield of erect-panicle varieties, the primary breeding goal is to develop long grain *geng/japonica* rice with the desired appearance qualities via the genetic improvement of grain shape (Xu and Chen 2016; Huang and Qian 2017). As pioneer research establishments, the China National Rice Research Institute (CNRRI) and Jiaxing Academy of Agricultural Sciences have developed Jiahe 218 and Jiahe 212 with a grain length to width ratio of 3.0 through the aggregation of *dep1* and *gs3* (Huang and Qian 2017), which provides a reference for the genetic improvement of grain shape in erect-panicle varieties. However, the Jiahe series rice varieties contain a large amount of tropical *japonica* background (Huang and Qian 2017), which is different from our research basing on the temperate *japonica* background. In the present study, YF47^*dep1*^-*gw8* grain exhibited the greatest length to width ratio of 2.2, comparing the best-known long grain high quality *japonica* rice varieties (the length to width ratio is generally more than 2.5) (Huang and Qian 2017), the grain shape of NILs in this article is still not ideal. We speculated that the simultaneous pyramiding of *GS3*/*TGW6* and *GW8* long type allele should be an effective way of developing erect-panicle *geng/japonica* rice with excellent appearance quality and high yield.

In summary, our results demonstrated that editing of grain shape genes was an effective approach to creating long-grain erect-panicle *geng/japonica* rice germplasm. Owning to both of the appearance quality and yield improvement, *GS3* and *TGW6* alleles can be applied directly for breeding long-grain shape *geng/japonica* rice, and editing *GW8* resulted in excellent appearance quality but low yield. Therefore, this gene would be difficult to use directly but can be considered as the core germplasm resource. All this work could provide the required germplasm and theoretical basis for breeding of high-yielding long-grain *geng/japonica* rice cultivars.

## Supplementary Information

**Additional file 1**. Materials and Methods

**Additional file 2: Supplemental Table 1**. The distribution of genotype on *GW8*, *GS3*, *GL7*, *qGL3* and *TGW6* derived from 96 rice germplasms in Liaoning province of China.

**Additional file 3: Supplemental Fig. 1**. Genotype analysis of *GW8*, *GS3*, *GL7*, *qGL3* and *TGW6* on YF47^*dep1*^ and 96 rice germplasms.

**Additional file 4: Supplemental Fig. 2**. The gene structures, target sequences for editing genes and vector map.

**Additional file 5: Supplemental Table 2**. Primers used to analyze target sequences and identify vectors.

**Additional file 6: Supplemental Table 3**. Primers used for Real-time PCR.

## Data Availability

The data sets supporting the results of this article are included within the article and its additional files.

## Abbreviations

*dep1*: *dense and erect-panicle 1*
NILs: Near isogenic lines
YF47^*dep1*^: Yanfeng 47
BR: Brassinosteroids
IAA: Indole acetic acid
ADPG: Adenosine diphosphate glucose
GBSS: Granule‐bound starch synthase
SSS: Soluble starch synthase
SBE: Starch branching enzyme
WT: Wild type
NEP: Number of effective panicles
NFGP: Number of filled grains per panicle
TGW: Thousand grain weight
