# Supplementary material for: Effects of Grain Shape Genes Editing on Appearance Quality of Erect-Panicle Geng/Japonica Rice
Source: Rice (N Y). 2021 Aug 10;14:74. doi: 10.1186/s12284-021-00517-5 (PMC8355294; doi:10.1186/s12284-021-00517-5)
Supplement: Supplementary file 1 — Additional file 1. Materials and Methods [file 12284_2021_517_MOESM1_ESM.doc]

**Materials and Methods**

**Plant materials**

The experimental materials were the following NILs with edited *GW8*, *GS3*, *GL7*, *qGL3* and *TGW6* genes in the background of YF47*^dep1^*: YF47*^dep1^*-*gw8*, YF47*^dep1^*-*gs3*, YF47*^dep1^*-*gl7*, YF47*^dep1^*-*qgl3* and YF47*^dep1^*-*tgw6*, with YF47*^dep1^* as the control. These lines were planted in the experimental research field of Liaoning Saline Alkali Land Utilization Institute (Panjin, Liaoning, 41.07°N; 122.03°E) during two consecutive growing seasons of 2019–2020. A randomized block design was used and three replicates were performed in plot areas of 20 m^2^ with row/plant spacing of 30 cm × 13.3 cm. The planting dates were 04/22/2019 and 04/20/2020, while the transplanting dates were 05/26/2019 and 05/24/2020, respectively. The overall inputs of N, P_2_O_5_ and K_2_O were 210 kg ha^-1^, 90 kg ha^-1^, and 60 kg ha^-1^, respectively, and all other field management was the same as that for local production.

**Detection of *gw8*, *gs3*, *gl7*, *qgl3* and *tgw6* genotype distribution in Liaoning province**

96 *japonica* rice varieties comprised 56 approved rice varieties (Number. 1-56) and 40 regional test strains (Number. 57-96) derived from Liaoning province, and the detection methods and primers selection referred to Sun et al., 2012 and Wang et al., 2012.

**Target design, vector construction and Agrobacterium transformation**


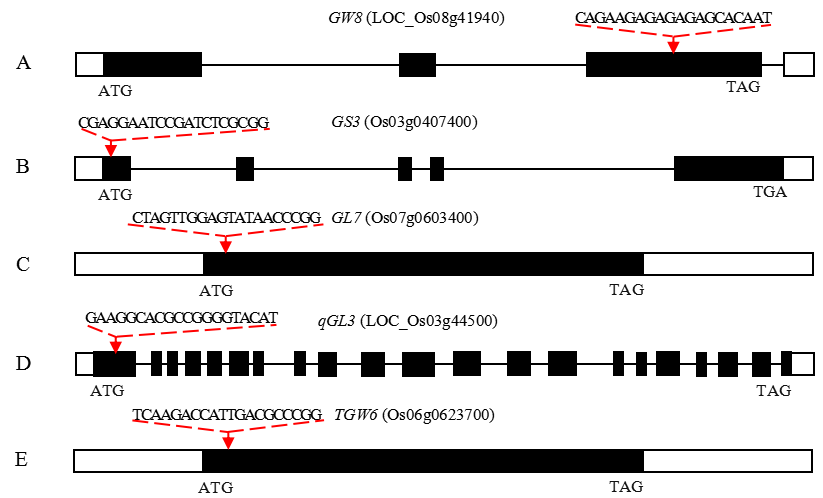
The gRNA targets were designed according to <http://crispr.dbcls.jp/>. The *GL7* target was designed from the adjacent negative regulator ([Os07g0603400](http://rapdb.dna.affrc.go.jp/viewer/gbrowse_details/irgsp1?name=Os07g0603400)), and the *GW8* target was designed from the miRNA156 region of the third exon. All other gene targets were on the first exon (Supplemental Fig. 2a). The adaptor (TGTGTG) was added to the 5ʹ end of the forward target sequence, and AC and CAAA were added to the 5ʹ and 3ʹ ends of the reverse sequence, respectively. The gRNAs were inserted into the Cas/gRNA plasmid using a vector construction kit (Cat#BGK03, Baige, China). The vector map is shown in Supplemental Fig. 2b. Agrobacterium transformation was carried out at Baige Biotechnology Co. Ltd.

**Determination of the mutation type and screening of plants with no vector**

The target sequences were analyzed in T_1_-T_2_ transgenic plants. For plants with homozygous mutations at the target, PCR was performed with primers of Cas9 sequences and hygromycin. The plants with no amplification of the target product were considered transgenic without vector insertion. The primers used are included in Supplementa Table 2.

**Determination of cell size and number, gene expression and hormone levels during glume development**

The cell size and number, gene expression and hormone levels were determined in the year 2020. Two days before heading, 30 glume samples were collected from the mid panicle of all tested lines, cut into cuboid shapes (1 mm × 3 mm) along the longitudinal center and fixed in 2.5% glutaraldehyde as previously described (Wang et al., 2012). The length, width and number of glume epidermal cells were determined using a scanning electron microscope (HATACHI Regulus 8100, Japan). The glume samples for gene expression and hormone levels testing were collected during the most vigorous period of glume development (when the young panicles were 8 cm long) (Li et al., 2011), and the expression of *DEP1* was investigated by Real-time PCR using the internal control designed by Song et al., 2007 (Supplemental Table 3). In addition, IAA and BR were extracted according to the methods of Bollmark (Bollmark et al.,1998), and the levels were determined using a commercial assay kit (Jiangsu Jingmei Biotechnology Co., Ltd., Yancheng, China) (Jiang et al., 2019).

**Determination of filling characteristics and enzyme activity during endosperm development**

The filling characteristics and enzyme activity were explored during endosperm development in 2020. Based on the methods described by Dong et al., 2008, 100 spikelets of similar size were selected and labelled for each line at the heading stage. During the 35 days after flowering, 10 labeled spikelets were collected every 5 days and kept. The samples were dried in an oven until they reached a constant weight, and the glumes were peeled off manually to measure the dry weight of the endosperm. The grain filling characteristics were analyzed using Richards’s equation (Zhu et al., 1988). As previously described (Wang et al., 2015), the endosperm was sampled at its most vigorous developmental stage (10 days after fertilization). The activity of the starch biosynthesis enzymes, ADPG, GBSS, SSS and SBE, was quantified using ELISA (Jiangsu Meimian Industrial Co. Ltd., Yancheng, China) according to Sun et al., 2019.

**Determination of grain shape, chalkiness degree and yield traits**

The measurements of grain shape and chalkiness degree were determined following harvest in the year 2020. Grains were harvested after they had reached the stage of yellow ripeness and stored at room temperature for 3 months. Thirty plump grains were selected randomly from each NIL to measure the grain length and width. After processing into polished rice, the chalkiness degree was assessed using an SC-E rice appearance quality analyzer (Wanshen, China). The investigations of effective panicle number, number of filled grains per panicle and thousand grain weight were performed using 10 plants for each replicate during the yellow ripeness stage (2019-2020). Production from a 1 m^2^ area was measured and the yield calculated under a moisture content of 14.5%.

**Supplemental References**

Bollmark M, Kubát B, Eliasson L (1998) Variations in endogenous cytokinincontent during adventitious root formation in pea cuttings. Journal of Plant Physiology 132:262–265

Dong MH, Zhao BH, Wu XZ, Chen T, Yang JC (2008) Difference in hormonal content and activities of key enzymes in the grains at different positions on a rice panicle during grain filling and their correlations with rice qualities. Scientia Agricultura Sinica 41(2):370–380 (in Chinese)

Jiang M, Liu YH, Li RQ, Zheng YC, Fu HW, Tan YY, Møller IM, Fan LJ, Shu QY, Huang JZ (2019) A suppressor mutation partially reverts the *xantha* trait via lowered methylation in the promoter of *genomes uncoupled 4* in rice. Front. Plant Sci 10:1003

Li YB, Fan CC, Xing YZ, Jiang YH, Luo LZ, Sun L, Shao D, Xu CJ, Li XH, Xiao JH, He YQ, Zhang QF (2011) Natural variation in *GS5* plays an important role in regulating grain size and yield in rice. Nature Genetics 43(12):1296–1269

Song XJ, Huang W, Shi M, Zhu MZ, Lin HX (2007) A QTL for rice grain width and weight encodes a previously unknown RING-type E3 ubiquitin ligase. Nature Genetics 39:623–630

Sun J, Liu D, Wang JY, Ma DR, Tang L, Gao H, Xu ZJ, Chen WF (2012) The contribution of intersubspecific hybridization to the breeding of super-high-yielding *japonica* rice in northeast China. Theoretical and Applied Genetics 125(6):1149–1157

Sun JH, Qiu C, Qian WJ, Wang Y, Sun LT, Li YS, Ding ZT (2019) Ammonium triggered the response mechanism of lysine crotonylome in tea plants. BMC Genomics 20:340

Wang SK, Li S, Liu Q, Wu K, Zhang JQ, Wang SS, Wang Y, Chen XB, Zhang Y, Gao CX, Wang F, Huang HX, Fu XD (2015) The *OsSPL16*-*GW7* regulatory module determines grain shape and simultaneously improves rice yield and grain quality. Nature Genetics 47(8):949–954

Wang SK, Wu K, Yuan QB, Liu XY, Zheng BL, Lin XY, Zeng RZ, Zhu HT, Dong GJ, Qian Q, Zhang GQ, Fu XD (2012) Control of grain size, shape and quality by *OsSPL16* in rice. Nature Genetics 44(8):950–954

Zhu QS, Cao XZ, Luo YQ (1998) Growth analysis on the process of grain filling in rice. Acta Agronomica Sinica 14(3):184–192(in Chinese)
