# Supplementary material for: Effects of Grain Shape Genes Editing on Appearance Quality of Erect-Panicle Geng/Japonica Rice
Source: Rice (N Y). 2021 Aug 10;14:74. doi: 10.1186/s12284-021-00517-5 (PMC8355294; doi:10.1186/s12284-021-00517-5)
Supplement: Supplementary file 2 — Additional file 2: Supplemental Table 1. The distribution of genotype on GW8, GS3, GL7, qGL3 and TGW6 derived from 96 rice germplasms in Liaoning province of China. [file 12284_2021_517_MOESM2_ESM.doc]

**Supplemental Table 1** The distribution of genotype on *GW8, GS3, GL7, qGL3* and *TGW6* derived from 96 rice germplasms in Liaoning province of China

| **Number** | **Variety (or line) names** | ***GW8*** | ***GS3*** | ***GL7*** | ***qGL3*** | ***TGW6*** |
| --- | --- | --- | --- | --- | --- | --- |
| 1 | Yanjing 456 | “NIP” type | “NIP” type | “NIP” type | “NIP” type | “NIP” type |
| 2 | Yanjing 933 | “NIP” type | “NIP” type | “NIP” type | “NIP” type | “NIP” type |
| 3 | Yanjing 431 | “NIP” type | “NIP” type | “NIP” type | “NIP” type | “NIP” type |
| 4 | Yanjing 1301 | “NIP” type | “NIP” type | “NIP” type | “NIP” type | “NIP” type |
| 5 | Yanjing 1302 | “NIP” type | “NIP” type | “NIP” type | “NIP” type | “NIP” type |
| 6 | Yanjing 1401 | “NIP” type | “NIP” type | “NIP” type | “NIP” type | “NIP” type |
| 7 | Shennong 625 | “NIP” type | “NIP” type | “NIP” type | “NIP” type | “NIP” type |
| 8 | Xiuqiudao 369 | “NIP” type | “NIP” type | “NIP” type | “NIP” type | “NIP” type |
| 9 | Shixindao 405 | “NIP” type | “NIP” type | “NIP” type | “NIP” type | “NIP” type |
| 10 | Meifengdao 336 | “NIP” type | “NIP” type | “NIP” type | “NIP” type | “NIP” type |
| 11 | Meifengdao 331 | “NIP” type | “NIP” type | “NIP” type | “NIP” type | “NIP” type |
| 12 | Tianyudao 18 | “NIP” type | “NIP” type | “NIP” type | “NIP” type | “NIP” type |
| 13 | Tianyudao 6 | “NIP” type | “NIP” type | “NIP” type | “NIP” type | “NIP” type |
| 14 | Yanjing 337 | “NIP” type | “NIP” type | “NIP” type | “NIP” type | “NIP” type |
| 15 | Yanjing 219 | “NIP” type | “NIP” type | “NIP” type | “NIP” type | “NIP” type |
| 16 | Yanjing 313 | “NIP” type | “NIP” type | “NIP” type | “NIP” type | “NIP” type |
| 17 | Danjing 24 | “NIP” type | “NIP” type | “NIP” type | “NIP” type | “NIP” type |
| 18 | Liaojing 1540 | “NIP” type | “NIP” type | “NIP” type | “NIP” type | “NIP” type |
| 19 | Yanjing 752 | “NIP” type | “NIP” type | “NIP” type | “NIP” type | “NIP” type |
| 20 | Yanjing 431 | “NIP” type | “NIP” type | “NIP” type | “NIP” type | “NIP” type |
| 21 | Tiejing 16 | “NIP” type | “NIP” type | “NIP” type | “NIP” type | “NIP” type |
| 22 | Panjing 968 | “NIP” type | “NIP” type | “NIP” type | “NIP” type | “NIP” type |
| 23 | Yangguangdao 63 | “NIP” type | “NIP” type | “NIP” type | “NIP” type | “NIP” type |
| 24 | Tiejing 1507 | “NIP” type | “NIP” type | “NIP” type | “NIP” type | “NIP” type |
| 25 | Beijing 1705 | “NIP” type | “NIP” type | “NIP” type | “NIP” type | “NIP” type |
| 26 | Fangjing 923 | “NIP” type | “NIP” type | “NIP” type | “NIP” type | “NIP” type |
| 27 | Danjing 14 | “NIP” type | “NIP” type | “NIP” type | “NIP” type | “NIP” type |
| 28 | Liaojing 2501 | “NIP” type | “NIP” type | “NIP” type | “NIP” type | “NIP” type |
| 29 | Danjing 16 | “NIP” type | “NIP” type | “NIP” type | “NIP” type | “NIP” type |
| 30 | Liaojing 1402 | “NIP” type | “NIP” type | “NIP” type | “NIP” type | “NIP” type |
| 31 | Tiejing 20 | “NIP” type | “NIP” type | “NIP” type | “NIP” type | “NIP” type |
| 32 | Beijing 1604 | “NIP” type | “NIP” type | “NIP” type | “NIP” type | “NIP” type |
| 33 | Shendao 171 | “NIP” type | “NIP” type | “NIP” type | “NIP” type | “NIP” type |
| 34 | Tiejing 17 | “NIP” type | “NIP” type | “NIP” type | “NIP” type | “NIP” type |
| 35 | Liaojing 237 | “NIP” type | “NIP” type | “NIP” type | “NIP” type | “NIP” type |
| 36 | Yujing 5 | “NIP” type | “NIP” type | “NIP” type | “NIP” type | “NIP” type |
| 37 | Hunjing 219 | “NIP” type | “NIP” type | “NIP” type | “NIP” type | “NIP” type |
| 38 | Beijing 1501 | “NIP” type | “NIP” type | “NIP” type | “NIP” type | “NIP” type |
| 39 | Liaojing 419 | “NIP” type | “NIP” type | “NIP” type | “NIP” type | “NIP” type |
| 40 | Fengmin 8 | “NIP” type | “NIP” type | “NIP” type | “NIP” type | “NIP” type |
| 41 | Jindao 109 | “NIP” type | “NIP” type | “NIP” type | “NIP” type | “NIP” type |
| 42 | Yanjing 662 | “NIP” type | “NIP” type | “NIP” type | “NIP” type | “NIP” type |
| 43 | Danjing 23 | “NIP” type | “NIP” type | “NIP” type | “NIP” type | “NIP” type |
| 44 | Yudao 17 | “NIP” type | “NIP” type | “NIP” type | “NIP” type | “NIP” type |
| 45 | Yanjing 468 | “NIP” type | “NIP” type | “NIP” type | “NIP” type | “NIP” type |
| 46 | Liaojing 1499 | “NIP” type | “NIP” type | “NIP” type | “NIP” type | “NIP” type |
| 47 | Shendao 505 | “NIP” type | “NIP” type | “NIP” type | “NIP” type | “NIP” type |
| 48 | Liaojing 1305 | “NIP” type | “NIP” type | “NIP” type | “NIP” type | “NIP” type |
| 49 | Jindao 107 | “NIP” type | “NIP” type | “NIP” type | “NIP” type | “NIP” type |
| 50 | Yanjing 765 | “NIP” type | “NIP” type | “NIP” type | “NIP” type | “NIP” type |
| 51 | Tianlongjing 213 | “NIP” type | “Minhui 63” type | “NIP” type | “NIP” type | “NIP” type |
| 52 | Liaojing 337 | “NIP” type | “NIP” type | “NIP” type | “NIP” type | “NIP” type |
| 53 | Danjing 20 | “NIP” type | “NIP” type | “NIP” type | “NIP” type | “NIP” type |
| 54 | Danjing 21 | “NIP” type | “NIP” type | “NIP” type | “NIP” type | “NIP” type |
| 55 | Liaojing 433 | “NIP” type | “NIP” type | “NIP” type | “NIP” type | “NIP” type |
| 56 | Daoyuan 16 | “NIP” type | “NIP” type | “Ping 13” type | “NIP” type | “NIP” type |
| 57 | Dan 1607 | “NIP” type | “NIP” type | “NIP” type | “NIP” type | “NIP” type |
| 58 | Dan 167 | “NIP” type | “NIP” type | “NIP” type | “NIP” type | “NIP” type |
| 59 | Dan 175 | “NIP” type | “NIP” type | “NIP” type | “NIP” type | “NIP” type |
| 60 | DDSZ-2 | “NIP” type | “Minhui 63” type | “NIP” type | “NIP” type | “NIP” type |
| 61 | DDSC-2 | “NIP” type | “NIP” type | “NIP” type | “NIP” type | “NIP” type |
| 62 | Yanjing 144 | “NIP” type | “NIP” type | “NIP” type | “NIP” type | “NIP” type |
| 63 | Yanjing 145 | “NIP” type | “NIP” type | “NIP” type | “NIP” type | “NIP” type |
| 64 | Huajing 1704 | “NIP” type | “NIP” type | “NIP” type | “NIP” type | “NIP” type |
| 65 | Huajing 1608 | “NIP” type | “NIP” type | “NIP” type | “NIP” type | “NIP” type |
| 66 | LDZ18-2 | “NIP” type | “NIP” type | “Ping 13” type | “NIP” type | “NIP” type |
| 67 | Liao 1758 | “NIP” type | “NIP” type | “NIP” type | “NIP” type | “NIP” type |
| 68 | Liao 802 | “NIP” type | “NIP” type | “NIP” type | “NIP” type | “NIP” type |
| 69 | Beijing R1 | “NIP” type | “NIP” type | “NIP” type | “NIP” type | “NIP” type |
| 70 | Beijing R2 | “NIP” type | “NIP” type | “NIP” type | “NIP” type | “NIP” type |
| 71 | Beijing R3 | “NIP” type | “NIP” type | “NIP” type | “NIP” type | “NIP” type |
| 72 | Beijing R4 | “NIP” type | “NIP” type | “NIP” type | “NIP” type | “NIP” type |
| 73 | Beijing R5 | “NIP” type | “NIP” type | “NIP” type | “NIP” type | “NIP” type |
| 74 | Beijing R6 | “NIP” type | “NIP” type | “NIP” type | “NIP” type | “NIP” type |
| 75 | Beijing R7 | “NIP” type | “NIP” type | “NIP” type | “NIP” type | “NIP” type |
| 76 | Beijing R8 | “NIP” type | “NIP” type | “NIP” type | “NIP” type | “NIP” type |
| 77 | Yanjing 114 | “NIP” type | “Minhui 63” type | “NIP” type | “NIP” type | “NIP” type |
| 78 | Shendao 100 | “NIP” type | “NIP” type | “NIP” type | “NIP” type | “NIP” type |
| 79 | Shendao 897 | “NIP” type | “NIP” type | “NIP” type | “NIP” type | “NIP” type |
| 80 | G305 | “NIP” type | “NIP” type | “NIP” type | “NIP” type | “NIP” type |
| 81 | G362 | “NIP” type | “NIP” type | “NIP” type | “NIP” type | “NIP” type |
| 82 | Liao 812 | “NIP” type | “NIP” type | “NIP” type | “NIP” type | “NIP” type |
| 83 | Liao 819 | “NIP” type | “NIP” type | “NIP” type | “NIP” type | “NIP” type |
| 84 | Huaxiangjing 2 | “NIP” type | “NIP” type | “NIP” type | “NIP” type | “NIP” type |
| 85 | Yongdaoxiang | “NIP” type | “Minhui 63” type | “NIP” type | “NIP” type | “NIP” type |
| 86 | Shendao 536 | “NIP” type | “NIP” type | “NIP” type | “NIP” type | “NIP” type |
| 87 | Yuandao 6 | “NIP” type | “NIP” type | “NIP” type | “NIP” type | “NIP” type |
| 88 | Yuandao 16 | “NIP” type | “NIP” type | “NIP” type | “NIP” type | “NIP” type |
| 89 | Liaojingxiang 2 | “NIP” type | “NIP” type | “NIP” type | “NIP” type | “NIP” type |
| 90 | Yanjing 488 | “NIP” type | “NIP” type | “NIP” type | “NIP” type | “NIP” type |
| 91 | Shennong 168 | “NIP” type | “NIP” type | “NIP” type | “NIP” type | “NIP” type |
| 92 | Shennong 922 | “NIP” type | “NIP” type | “NIP” type | “NIP” type | “NIP” type |
| 93 | Jindao 919 | “NIP” type | “NIP” type | “NIP” type | “NIP” type | “NIP” type |
| 94 | Tianlongjing 46 | “NIP” type | “NIP” type | “NIP” type | “NIP” type | “NIP” type |
| 95 | Jindao 501 | “NIP” type | “NIP” type | “NIP” type | “NIP” type | “NIP” type |
| 96 | Yujingxiang 6 | “NIP” type | “NIP” type | “NIP” type | “NIP” type | “NIP” type |

NIP, Nipponbare, “Minhui 63” type and “Ping 13” type represent long grain type for *GS3* and *GL7* loci, respectively, and “NIP” type represent short and round grain type for all the above five locus.
