# Supplementary material for: Effects of Grain Shape Genes Editing on Appearance Quality of Erect-Panicle Geng/Japonica Rice
Source: Rice (N Y). 2021 Aug 10;14:74. doi: 10.1186/s12284-021-00517-5 (PMC8355294; doi:10.1186/s12284-021-00517-5)
Supplement: Supplementary file 3 — Additional file 3: Supplemental Fig. 1. Genotype analysis of GW8, GS3, GL7, qGL3 and TGW6 on YF47dep1 and 96 rice germplasms. [file 12284_2021_517_MOESM3_ESM.doc]

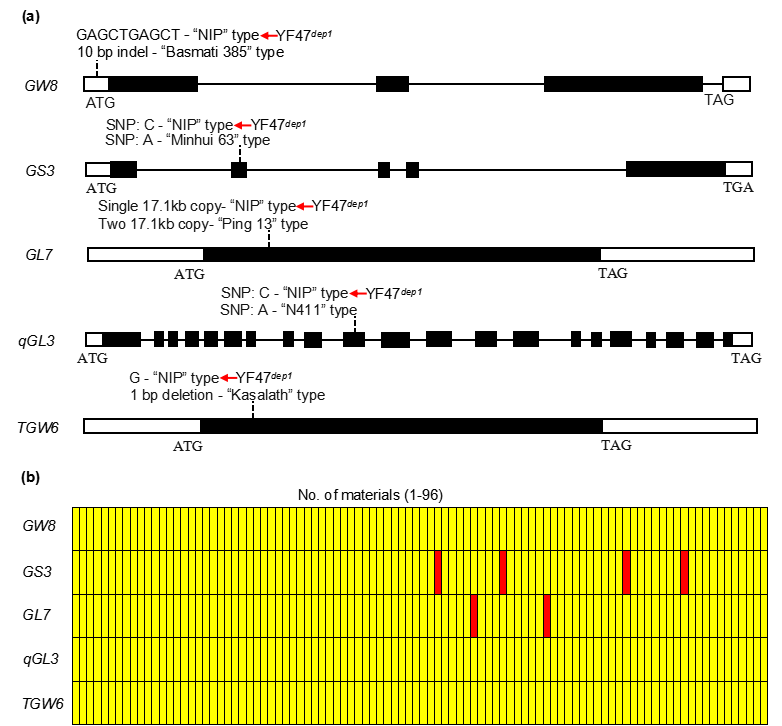


**Supplemental Fig. 1** Genotype analysis of *GW8*, *GS3*, *GL7*, *qGL3* and *TGW6* on YF47*^dep1^* and 96 rice gremplasms. **a,** Genotype analysis of *GW8*, *GS3*, *GL7*, *qGL3* and *TGW6* on YF47*^dep1^*. NIP, Nipponbare. **b,** Genotype analysis of *GW8*, *GS3*, *GL7*, *qGL3* and *TGW6* on 96 rice gremplasms, yellow region represents short and round grain type (“NIP” type), and red region represents long grain type (“Minhui 63” type and “Ping 13” for *GS3* and *GL7* loci, respectively).
