# Supplementary material for: Effects of Grain Shape Genes Editing on Appearance Quality of Erect-Panicle Geng/Japonica Rice
Source: Rice (N Y). 2021 Aug 10;14:74. doi: 10.1186/s12284-021-00517-5 (PMC8355294; doi:10.1186/s12284-021-00517-5)
Supplement: Supplementary file 4 — Additional file 4: Supplemental Fig. 2. The gene structures, target sequences for editing genes and vector map. [file 12284_2021_517_MOESM4_ESM.doc]

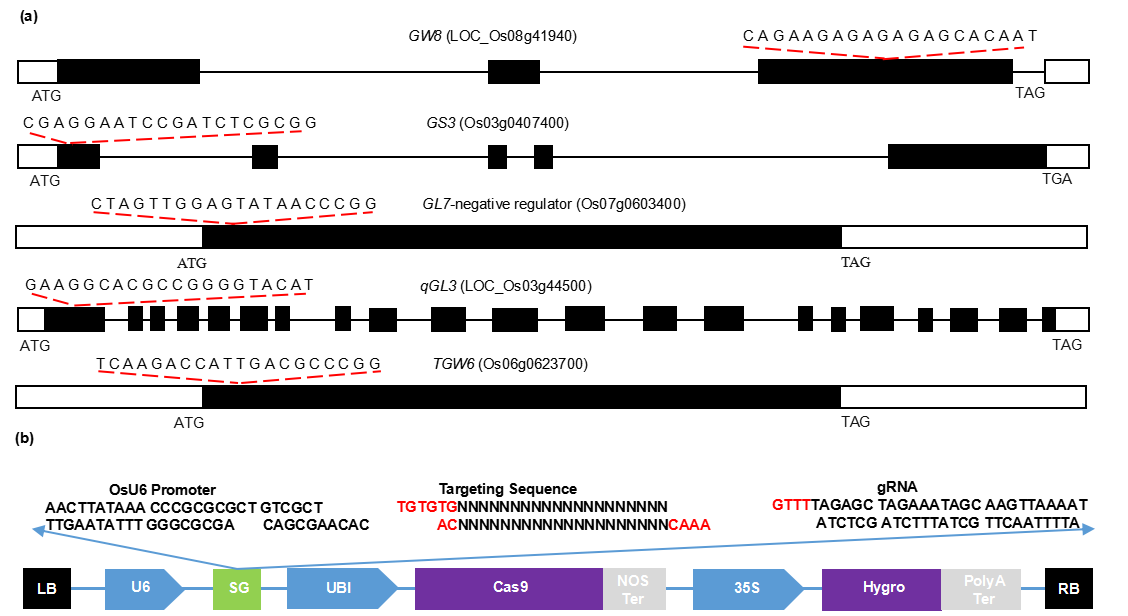


**Supplemental Fig. 2** The gene structures, target sequences for editing genes and vector map. **a,** The position and target sequences of *GW8*, *GS3*, *GL7*, *qGL3* and *TGW6*. The black boxes and lines represent exons and introns, respectively. **b,** Vector map of Cas9/gRNA. LB, Left border of T-DNA; U6, Rice U6 promoter; SG, sgRNA; UBI, UBI promoter; Cas9, Optimized Cas9; NOSTer, NOS terminator; 35S, CaMV 35S promoter; Hygro, Hygromycin selection marker; PolyA Ter, PolyA terminator; RB, Right border of T-DNA.
