# Supplementary material for: Effects of Grain Shape Genes Editing on Appearance Quality of Erect-Panicle Geng/Japonica Rice
Source: Rice (N Y). 2021 Aug 10;14:74. doi: 10.1186/s12284-021-00517-5 (PMC8355294; doi:10.1186/s12284-021-00517-5)
Supplement: Supplementary file 5 — Additional file 5: Supplemental Table 2. Primers used to analyze target sequences and identify vectors. [file 12284_2021_517_MOESM5_ESM.doc]

**Supplemental Table 2** Primers used to analyze target sequences and identify vectors

| Primer name | Primer sequence (5'-3') | Produce size (bp) |
| --- | --- | --- |
| *GW8*-F | GTTGGCTCCACCTCTGAC | 359 |
| *GW8*-R | CCAGAGATGAGAGGCTGC |
| *GS3*-F | GGAGAAGAAAAGAGCGGC | 559 |
| *GS3*-R | GGGAAAAACTTACAATGA |
| *GL7*-F | TTGGCTTGGTTCGCTGAT | 626 |
| *GL7*-R | GCCGCCGACTGTATCAAT |
| *qGL3*-F | GGCTTAGCGAGCGAGACC | 393 |
| *qGL3*-R | ACACGACCAATCGACATT |
| *TGW6*-F | TGCTCCTCCAATCTCTCA | 581 |
| *TGW6*-R | ACCAACTCGCATCAATCC |
| *Cas9*-F | CACCATCTACCACCTGAGAA | 371 |
| *Cas9*-R | CGAAGTTGCTCTTGAAGTTG |
| *Hyg*-F | GCTGTTATGCGGCCATTGTC | 350 |
| *Hyg*-R | GACGTCTGTCGAGAAGTTTC |
