# Supplementary material for: Effects of Grain Shape Genes Editing on Appearance Quality of Erect-Panicle Geng/Japonica Rice
Source: Rice (N Y). 2021 Aug 10;14:74. doi: 10.1186/s12284-021-00517-5 (PMC8355294; doi:10.1186/s12284-021-00517-5)
Supplement: Supplementary file 6 — Additional file 6: Supplemental Table 3. Primers used for Real-time PCR. [file 12284_2021_517_MOESM6_ESM.doc]

**Supplemental Table 3 Primers used for Real-time PCR**

| Primer name | Primer sequence (5'-3) | Produce size (bp) |
| --- | --- | --- |
| *DEP1*-F (RT-PCR) | AGCCCGTTTCTCGTTCTG | 202 |
| *DEP1*-R (RT-PCR) | TTGAGGCACCTTGGTCTTT |
| Action-F | GACTCTGGTGATGGTGTCAGC | 200 |
| Action-R | GGCTGGAAGAGGACCTCAGG |
